# *In situ* biaxial loading and multi-scale deformation measurements of nanostructured materials at the CoSAXS beamline at MAX IV Laboratory

**DOI:** 10.1107/S1600576723005034

**Published:** 2023-06-30

**Authors:** Pablo Mota-Santiago, Jonas Engqvist, Stephen Hall, Roberto Appio, Maxime Maghe, Gautham Sathikumar, Matti Ristinmaa, Tomás S. Plivelic

**Affiliations:** a MAX IV Laboratory, Lund University, 118, Lund 22100, Sweden; b Division of Solid Mechanics, Lund University, 118, Lund 22100, Sweden; c Institute for Frontier Materials, Deakin University, 75 Pigdons Road, Geelong, Victoria 3216, Australia; Brazilian Synchrotron Light Laboratory, Brazil

**Keywords:** Biaxial mechanical testing, soft matter, small-angle X-ray scattering, SAXS, wide-angle X-ray scattering, WAXS

## Abstract

Characterization of the mechanical response of polymers and composite materials relies heavily on the macroscopic stress–strain response in uniaxial tensile configurations. To provide representative information, the deformation process must be homogeneous within the gauge length, which is a condition that is rarely achieved due to stress concentration or inhomogeneities within the specimen. In this work, the development of a biaxial mechanical testing device at the CoSAXS beamline at MAX IV Laboratory is presented. The design facilitates simultaneous measurement of small- and wide-angle X-ray scattering (SAXS/WAXS), allowing assessment of the microstructural configuration before, after and during the continuous deformation process at multiple length scales. The construction also supports multiple deformation conditions, while guaranteeing stability even at high loads. Furthermore, the mechanical experiments can be complemented with spatially resolved mesoscopic surface deformation measurements using 3D-surface digital image correlation (DIC). Polycarbonate (PC) was used to demonstrate the varied material response to multi-axial deformation, as PC is isotropic with a high glass transition temperature (∼150°) and high strength. As a result, a clear correlation between full-field methods and the microstructural information determined from WAXS measurements is demonstrated. When a uniaxial load is applied, homogeneous strain regions could be observed extending perpendicular to the applied load. When a secondary axial load was added (biaxial mode), it was observed that high strain domains were created near the centre of the sample and at the boundaries after yield. With increased strain, the deformation in the main deformation direction also increases. Mechanical reliability was demonstrated by carrying out static loading of polyacrylonitrile-based carbon fibre (CF) bundles. As a result, the nonlinear stiffening behaviour typically observed in CFs was seen, while no evidence of the creation of new voids during loading was observed. The results support the reliability and broad applicability of the developed technique.

## Abstract 2

A novel biaxial tensile tester designed to work in combination with 2D synchrotron-based scanning small- and wide-angle X-ray scattering and digital image correlation is described. The available biaxial modes and a proposed sample geometry are discussed.

## 1. Introduction

Traditionally, the characterization of the mechanical response of nanostructured and microstructured materials has been addressed mainly by the macroscopic force–displacement (or equivalently stress–strain) curves from uniaxial tensile or compression tests (Kiwabara, 2014 ▸). Although these curves can be easily obtained, the practical use of the macroscopic load–displacement response alone is often limited since the material localizes, resulting in a non-uniform deformation field.

Determination and prediction of the mechanical properties and damage accumulation in polymers, colloidal materials, biomaterials and composite materials have been widely explored in the past two decades, as they play a key role in the design and manufacture of novel components in several industries (Stasiak *et al.*, 2011 ▸; Meek & Penumadu, 2021 ▸). Nevertheless, to predict the material performance, it is necessary to take into consideration the microstructure of the material (Pathan *et al.*, 2019 ▸). In real applications, materials are often subjected to multi-axial deformation with complex load history, and an experimental investigation should aim to mimic these conditions as closely as possible (Kiwabara, 2014 ▸). Multi-axial deformation can be achieved by, for example, biaxial loading where the material is deformed in two perpendicular directions independently (Johlitz & Diebels, 2011 ▸; Engqvist *et al.*, 2016 ▸).

The orientation, dispersion and structural changes of polymeric materials and nanocomposites that a biaxial stretching process can induce have been shown to be beneficial for enhancing mechanical, optical and thermal properties of such systems (Abu-Zurayk *et al.*, 2010 ▸). Biaxially stretched materials, when compared with uniaxially stretched ones, exhibited more balanced mechanical properties and thermal shrinkage along the machine and transverse directions (Liu *et al.*, 2019 ▸). Biaxial stretching could also provide the ability to better disperse nanofillers in the polymeric matrix without using compatibilizers, which would be advantageous for the packaging industry (Xiang *et al.*, 2015 ▸). Furthermore, the use of biaxially oriented polymer nanocomposites in many fields has not been widely studied.

Biaxial stretching is not only an advanced film manufacturing process but also a deformation mode in other processing methods such as blow film extrusion and thermoforming (Portale *et al.*, 2013 ▸). Although sequential biaxial stretching and simultaneous biaxial stretching (or the combination of the two) have been used to fabricate various homopolymers and polymer nanocomposite films, there is still potential for optimization. Polyolefin and polyvinyl films are widely used due to their mechanical performance, low weight, thermal stability and low manufacturing cost. The post-stretching processing of polymer films involves the rapid evolution of multi-scale structures where temperature and strain rate are key factors affecting the structural transformation dynamics (Emblem, 2012 ▸; Sastri, 2022 ▸; Feng *et al.*, 2021 ▸). The structure, orientation degree and surface morphology of polymers can be changed by adjusting the stretching ratio of the biaxial stretching to enhance the strength and tensile modulus in the oriented direction (Soon *et al.*, 2012 ▸). Because the most common fabrication involves a melt state, structural orientation is not always maintained and, thus, special design and optimization of the drawing temperature, speed and draw ratios are required (Kong *et al.*, 2019 ▸).

Due to the complex morphology of many modern materials which involves multiple length scales, an accurate description of the mechanical behaviour requires multi-scale modelling in a broader range, ideally from the atomic scale to the macroscopic level. The small- and wide-angle X-ray scattering (SAXS/WAXS) techniques are ideal to describe submicrometre structural details, whereas full-field measurement techniques, such as digital image correlation (DIC), can be used to provide macroscopic materials parameters and the deformation process map for the entire sample (Urban *et al.*, 2021 ▸). DIC is based on tracking individual material points which allows the local deformation to be extracted. Using full-field measurements combined with scanning X-ray scattering over a large specimen area and in short exposure times, the experiments can be performed under inhomogeneous conditions. As such, the outcome of the experiments can provide much richer information about the spatial and temporal material behaviour compared with macroscopically (assumed) homogeneous tests.

Thus, the combination of *in situ* or X-ray techniques with multi-scale experiments can provide the information needed to develop physically sound models that accurately describe the mechanical behaviour of composites.

Carbon fibres (CFs) are well known for their high tensile strength, high modulus and relatively low density. Their microstructure typically exhibits fibrillated structures, formed by small coherent domains or crystallites aligned preferentially towards the fibre axis, amorphous regions, and a dispersion of defects or voids. Their high elastic modulus arises from the orientation of the graphitic planes within a few degrees from the fibre axis, and thus, the correlation between mechanical properties and the microstructure is evident.

In this work, the development of a biaxial mechanical testing device at the CoSAXS beamline at MAX IV Laboratory is presented. The mechanical reliability of the tensile tester was demonstrated by carrying out static loading of polyacrylonitrile (PAN)-based CF bundles. As a result, we observed and quantified the typically observed nonlinear stiffening behaviour using WAXS. Analysis of the SAXS scattering patterns showed no evidence of the creation of new voids during the loading process. The results support the reliability and broad applicability of the developed technique.

We also present two examples of different material response to multi-axial deformations. Polycarbonate (PC) is a glassy amorphous polymer at room temperature with high impact strength. When applying uniaxial load on the material, we could observe homogeneous strain regions extending perpendicular to the applied load. When adding a secondary axial load (biaxial mode), we observed the creation of domains, not only near the centre of the sample but also at the sample boundaries. With increased strain, the deformation also increased in the main deformation direction. A clear correlation between the microstructural information provided by DIC and the microstructure analysis provided by WAXS was observed.

## 2. Experimental

### 2.1. Biaxial mechanical testing and control system

**Figure 1:**
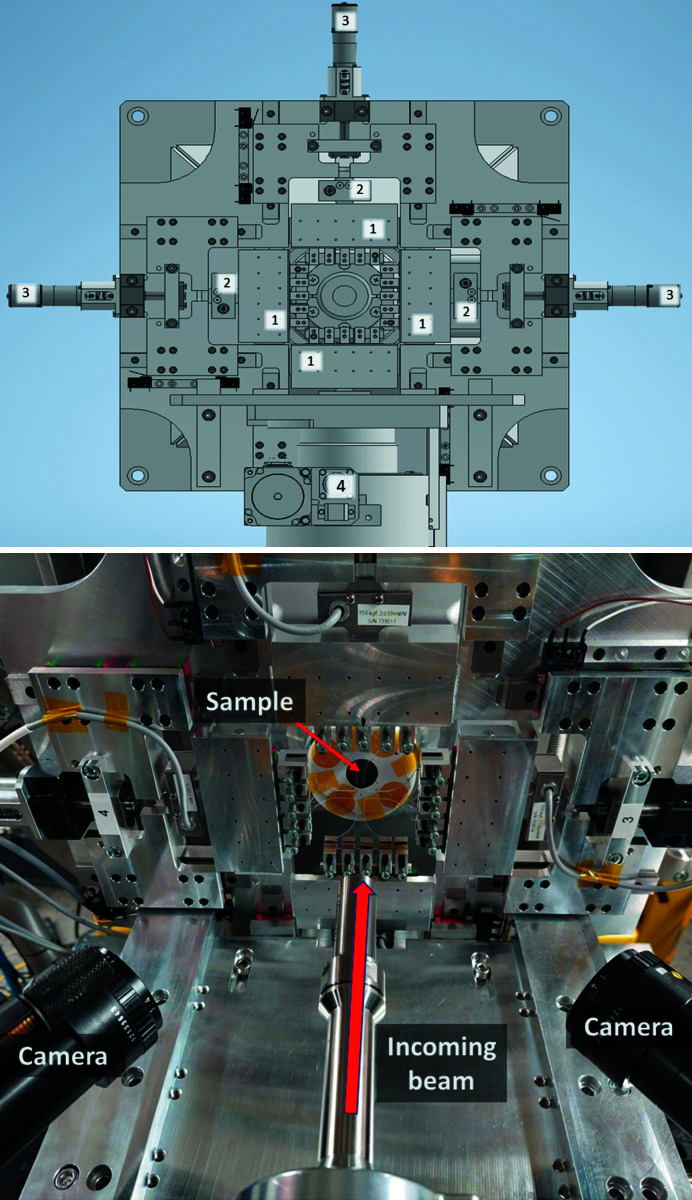
Biaxial setup depicting the main elements: (1) four movable grips, (2) four load cells and (3) four independent stepper motors, mounted on a high load *X*–*Y* stage (4) with position feedback provided by absolute encoders for fast and high-precision positioning.

The biaxial tensile tester design is based on a previous design by Engqvist *et al.* (2016 ▸), optimized for *in situ* loading. Special care has been taken to minimize the distance from the sample to the nose cone of the in-vacuum flight tube and thus maximize the probable detection area by the WAXS detector. The device consists of four arms. Each arm is composed of five small grips fitted with ball bearings for free lateral displacement, coupled to a load cell fixed to a carrier that translates by a stepper motor. The load cells can be exchanged between two resolutions (100 and 1500 N, respectively) for experiments requiring different sensitivities. The four arms are mounted in-plane and perpendicular to each other, as shown in Fig. 1 ▸(*a*). The fitting of the biaxial loading tester at the experimental table of the CoSAXS beamline, the relative positioning with respect to the incident beam and the positioning of the two DIC cameras are shown in Fig. 1 ▸(*b*). Each arm can be controlled autonomously, making operation along two independent stretching directions accessible with displacement rates between 0.02 and 15 mm min^−1^. The minimum distance between grips corresponds to 75–80 mm, permitting a travel distance of 20 mm per grip (*i.e.* 40 mm per axis) for large strain field coverage along two axes. The setup is mounted on a high-load-capacity *X*–*Y* translation stage with encoders for fast and accurate positioning.

The control system has been implemented in *LabView* for fast recording of the displacement and analogue readout from each stepper motor and load cell. Integration into the control system of the beamline occurs through an in-house *LabView2Tango* module. In this modality, the biaxial loading tester becomes a Tango device where the motor displacement and load cell readout values are treated as attributes, facilitating the recording of the experimental parameters by the beamline acquisition system. The control system allows control of the biaxial loading tester even without communication to the beamline control system (offline mode). The offline modality offers the user the possibility to carry out experiments prior to beam time access or at other beamlines and facilities. The control system has been designed to support monotonic and cyclic operation.

### 2.2. SAXS–WAXS measurements

**Figure 2:**
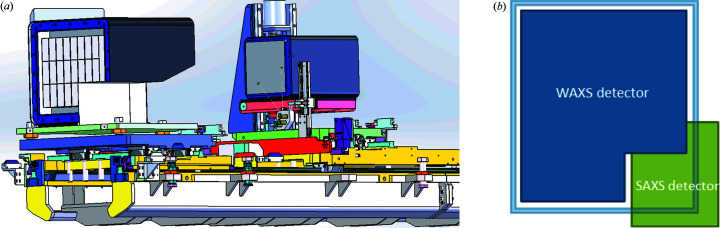
(*a*) SAXS and WAXS detector carriers inside the evacuated vessel of the CoSAXS beamline. (*b*) Schematic of the effective detection area layout in the SAXW/WAXS configuration.

Simultaneous SAXS and WAXS measurements were carried out at the CoSAXS beamline at MAX IV Laboratory, Sweden (Plivelic *et al.*, 2019 ▸). SAXS measurements were performed with an Eiger2 4M detector and WAXS measurements with an L-shaped Pilatus3 2M detector (both systems from Dectris AG). Both detectors are in-vacuum compatible and are placed inside the evacuated vessel operating at 0.001 mbar (see details in Fig. 2 ▸).

The measurements were carried out with a monochromatic X-ray beam of 18 keV. The beam cross section for the measurements at the sample position was 135 × 135 µm [full width at half-maximum (FWHM), horizontal × vertical]. A total area of 4 × 6 mm (horizontal × vertical) was scanned using a mesh scan in serpent trajectory with a step size of 0.5 mm in both directions and an exposure time of 1 s per position. The SAXS and WAXS detectors were positioned at 4 and 0.5 m from the sample, respectively, and provide a combined and continuous *q* range from 4 × 10^−3^ Å^−1^ up to 2.8 Å^−1^ [the scattering vector magnitude *q* is defined as 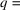 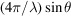 , 2θ is the scattering angle and λ is the wavelength].

For material analysis, two frameworks were developed. For PC, the analysis of the characteristic scattering patterns was carried out by reducing the 2D scattering pattern to 1D scattering intensity against scattering vector magnitude curves using the Python-based *azint* library developed at MAX IV Laboratory (available at https://github.com/maxiv-science/azint). Because the correlation between the local structure of PC and the WAXS data was already known, the extracted 1D scattering intensity was numerically fitted to identify the position of each contribution. After deconvolution of the WAXS signal, integration of the 2D scattering pattern centred at the main signal was carried out along the azimuthal direction. Under load, the azimuthal scattering curve provided information on preferred orientation, which was then numerically fitted using a Gaussian function. From the numerical results two directions were determined, parallel and perpendicular to the applied tension. From the azimuthal information, the SAXS and WAXS 2D patterns were decomposed along three orthogonal directions: (i) along the direction parallel to the tension, (ii) perpendicular to the applied tension and (iii) along the azimuthal direction. For CF, because the bundle was mounted parallel to the horizontal direction, the decomposition of the 2D SAXS and WAXS scattering patterns was done along three orthogonal directions, along the horizontal, vertical and azimuthal directions. For PC, the specimens were scanned around the geometrical centre of the sample by an area of 4 × 6 mm (horizontal × vertical) with a step resolution of 0.5 mm in both directions.

The SAXS and WAXS measurements were recorded during continuous loading. The image acquisition for DIC was synchronized with the detector triggering for optimal correlation between the strain field and the corresponding 2D scattering signals.

### 2.3. Materials and methods

The samples used in the dynamic load experiments were prepared from 1 mm-thick commercial PC. For the static loading experiments in uniaxial configuration mode, we tested CF bundles produced at the Research Line at the Carbon Nexus Research Centre, Deakin University, Australia (Maghe *et al.*, 2016 ▸). Fibres were manufactured using a commercial PAN-based precursor from Hexlan (Japan), resulting in standard modulus grade (ρ = 1.8 g cm^−3^ and modulus ≃ 240 GPa). Small bundles were prepared, with a tow size of 1k and a cross section of 0.8 mm^2^.

**Figure 3:**
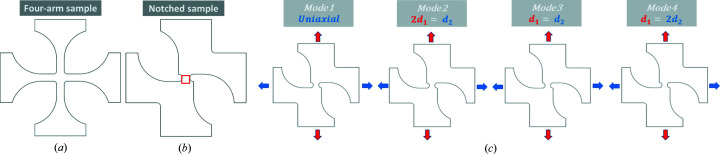
(*a*) Four-arm geometry – or cruciform geometry – typically used in biaxial stress–strain tests (Kiwabara, 2014 ▸). (*b*) Suggested variant to the cruciform geometry with symmetric notches. The red square highlights the region of interest during scanning. (*c*) The four modes of biaxial deformation under analysis. The uniaxial mode can be achieved by unclamping the specimen from the horizontal grips. Modes 2, 3 and 4 represent different displacement rates between the two axes of freedom.

Fig. 3 ▸(*a*) shows the cruciform geometry typically used for measuring stress–strain curves in biaxial tension (Kiwabara, 2014 ▸). The cruciform geometry has been previously adopted for the determination of contours of plastic behaviour. In our case, when using samples fabricated with this geometry, the deformation occurred at one of the arms (not shown).

To address the random occurrence of strain concentration outside of the region of interest, we proposed the geometry depicted in Fig. 3 ▸(*b*). It consists of two symmetrical arms on each end and symmetrical notches at the central position, creating a separation of 5 mm at the narrower section. The geometry favours a shearing component along the horizontal axis during the deformation process. In Fig. 3 ▸(*b*) the region of interest is highlighted where the deformation and concentration of strain is expected to occur.

Fig. 3 ▸(*c*) shows the different loading experiments, aiming to observe different material responses. Mode 1 is comparable to uniaxial loading as the displacement occurs along the sample axis (vertical direction) while keeping the lateral sides of the specimen unclamped from the corresponding grips. Due to the proposed geometry, we expect to observe a shearing component to the deformation process. Mode 2 is the first deformation experiment considering a 2:1 ratio between the axial displacement and the horizontal direction. We expect to induce elongation along the horizontal direction while also increasing the shearing component in the horizontal direction. Mode 3 considers a 1:1 displacement ratio between the axial and horizontal directions, *i.e.* an equivalent displacement along each axis of deformation. The last mode, mode 4, aims to study the structural deformation with a significant shearing component by changing the displacement ratio between the axial and horizontal directions to 1:2.

## 3. Results and discussion

**Figure 4:**
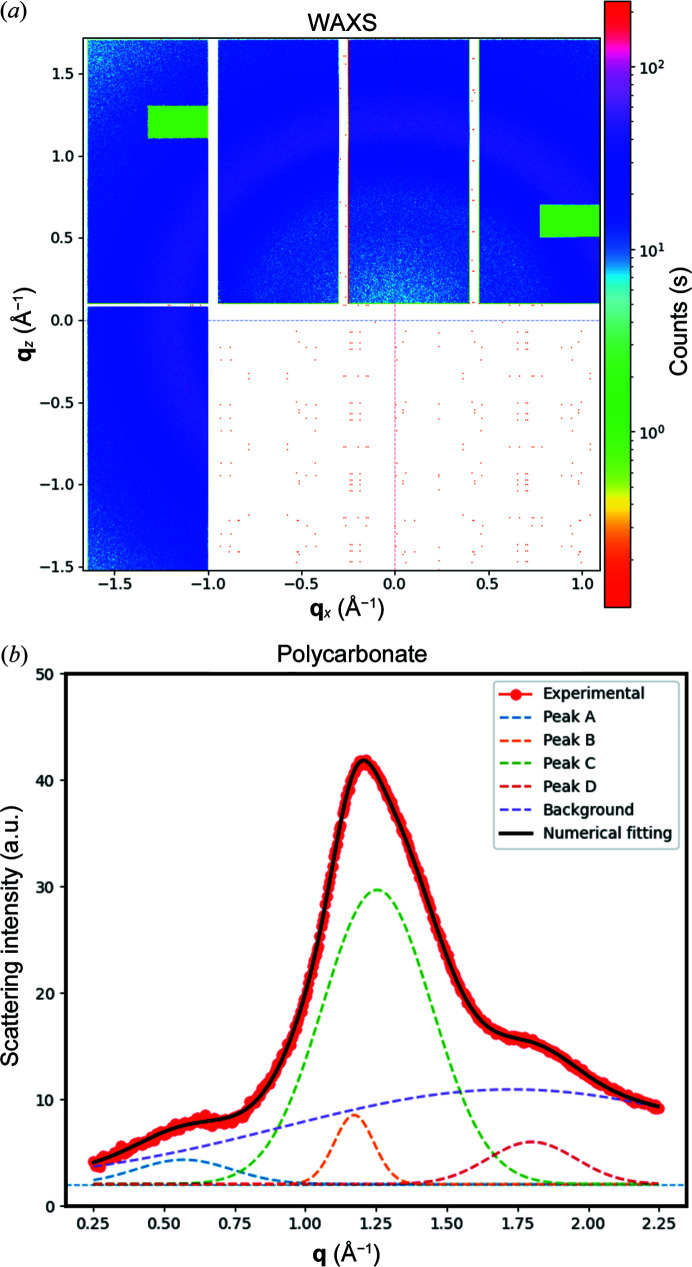
(*a*) WAXS 2D scattering pattern from pristine PC, two dotted lines have been added to indicate the beam centre. (*b*) Extracted scattering intensity from WAXS measurements of undeformed PC (red dotted line). The Gaussian contribution due to the correlation between consecutive C groups (peak A – dashed blue line), the distance between neighbouring chains (peak B – dashed orange line), the correlation between entities along the chain (peak C – green dashed line), and the inter- and intra-molecular correlations (peak D – dark-red dashed line) are plotted against the experimental measurements. The combination of the four contributions is shown as a dark line to demonstrate the agreement with the experimental data.

PC is an amorphous, transparent polymer with a high glass transition temperature (*T*_g_ ≃ 150) and impact strength that exhibits a typical molecular structure characterized by random coils. However, it can exhibit twin issues of chain conformation and local interchain correlations of length scales within 1–50 Å, where WAXS is of great relevance (Windle, 1985 ▸). Fig. 4 ▸(*a*) shows the 2D WAXS pattern of PC before loading. The presence of the characteristic diffuse halo is evident in PC while no preferred orientation is seen.

In Fig. 4 ▸(*b*), the scattering intensity from pristine PC is plotted. From the figure, three characteristic features associated with the PC chains can be identified, a diffuse shoulder around 1.8 Å^−1^, a small peak near 0.5 Å^−1^ and the main scattering contribution featured around 1.2 Å^−1^. The contribution at 1.8 Å^−1^ is commonly observed in main-chain aromatic polymers. Preferentially aligned PC materials have been observed to align towards 45°, in line with the conformation of the PC chain where virtual bonds make an angle between 30 and 35° with the main-chain axis (Windle, 1985 ▸). The small peak at 0.5 Å^−1^ is characteristic for PC, where the chains tend to orient parallel to the draw direction or along the meridional direction under load, and its position correlates well with the structural short-range order (Windle, 1985 ▸; Schubach & Heise, 1986 ▸; Engqvist *et al.*, 2016 ▸).

The principal contribution at 1.2 Å^−1^ exhibits a slight asymmetry that becomes more evident under load, suggesting the presence of two contributions. The weakest contribution has been attributed to the distance between neighbouring chains and is centred around 1.17 Å^−1^, resulting in a *d* spacing of 0.58 nm ( 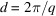 ). The principal contribution is centred at 1.25 Å^−1^ and has been linked to correlations between the different carbon groups along the main chain (Engqvist *et al.*, 2016 ▸). The halo has been observed to show a very strong orientation perpendicular to the draw direction or applied load.

The deconvolution of the experimental WAXS scattering intensity is presented in Fig. 4 ▸(*b*) considering the interpretation described above. Therefore, it is evident that under load the local structure at short range shows reorientation of the PC chains together with variations in the bond length due to the applied strain field.

As the principal local microstructure features show a direct correlation between orientation and the applied load, the degree of orientation at the nanometre scale can be quantified by approximating the FWHM of the scattering intensity along the azimuthal direction at each corresponding peak position. Using a Gaussian distribution for fitting the data, the orientation index (OI) is defined as

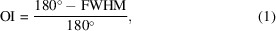

where a value closer to 1 represents a fully oriented system and a value of 0 corresponds to an isotropic network.

**Figure 5:**
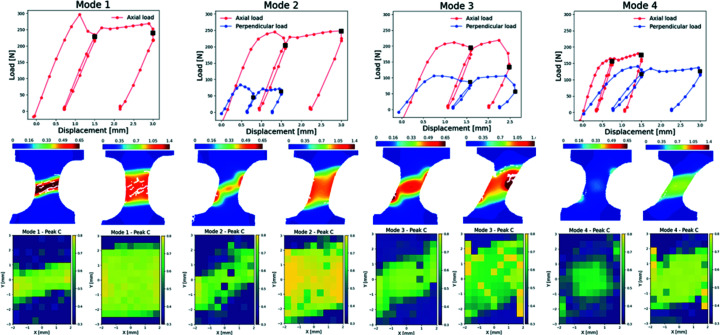
Force versus displacement curves for each mechanical test proposed. Full-field images are given that correspond to the maximum displacement at each load cycle for each deformation mode (dark squares on the force versus displacement curves). Spatially resolved orientation index from peak C under the same conditions as the DIC.

Fig. 5 ▸ shows the force-displacement curve measured by the biaxial tensile device for the four biaxial deformation modes proposed. For the first mode (uniaxial deformation), the behaviour agrees with previously observed uniaxial deformation of PC (Engqvist *et al.*, 2016 ▸). The curve is characterized by a monotonic increase in the force, after which the force suddenly drops before the first unloading process occurs. During the unloading process a permanent deformation was evident as the specimen did not return to its original dimensions. For the following loading cycle, the force increases monotonically, reaching a local maximum value but followed by a smooth decrease that rapidly transforms into a plateau and then increases at a low rate, suggesting strain-hardening of the material.

For the first biaxial mode, where the axial displacement is larger than the horizontal, we can observe a very similar behaviour to the uniaxial mode except for small differences. Among the differences, the material does not experience a force as high as for pure biaxial loading and exhibits a smoother transition between the maximum force, the plateau region and the strain hardening. Along the horizontal direction, the force versus displacement curve resembles that of the vertical mode but at a fraction of the force exerted on the specimen.

When the deformation rate is equal along the axial and horizontal directions, the material does not reach the maximum force value before the slight drop. Instead, it appears to be a very smooth transition before the first unloading step. Afterwards, the material reaches the maximum value in the middle of the loading process. Along the axis direction it appears to show a strain hardening behaviour, while along the horizontal direction, the effect is hindered. This is probably because of the shearing component along the horizontal direction becoming more relevant to the process.

For the fourth deformation mode, predominantly along the horizontal direction, the force versus displacement curve shows a very smooth transition between the local maximum force and the unloading of the first loading cycle. Afterwards, a plateau followed by a strain hardening behaviour is observed along both directions.

The DIC analysis shows the evolution of the major (tensile) stretch fields (Engqvist *et al.*, 2014 ▸, 2016 ▸). Fig. 5 ▸ shows the deformation fields when the sample is at the maximum displacement for each loading cycle (shown as dark squares on the corresponding force versus displacement curves). The uniaxial results show that the deformation of the sample occurs at the central gap of the specimen for the first cycle. At the end of the second cycle, high-deformation regions are formed near the edges of the pre-notched boundaries. The localization of high deformation at the edges demonstrates the presence of high-strain zones. High-strain zones can promote and initiate failure of the material that can propagate, finally leading to failure of the specimen.

For the three biaxial modes (modes 2 to 4), the strain developed an odd distribution along the centre of the sample at the maximum displacement in the first loading cycle. The strain distribution appears to align towards the resulting strain applied by the corresponding biaxial mode. Note that the strain concentration near the centre of the specimen was higher for mode 3, followed by mode 2 and mode 4. The trend observed in mode 4 is that the strain distribution during the two loading cycles is the lowest with respect to the other two biaxial modes. That is clearly observed when reaching the maximum displacement conditions. DIC data show a lower strain distributed almost homogeneously along the middle region of the specimen. This could be the result of shearing being the main deformation process. In mode 3, at the longest displacement conditions we observe the formation of an extended high-stress region at one of the boundaries of the specimen. The stress profile does not seem to follow the distribution observed in the first loading cycle, where a slightly higher stress concentration was observed at the opposite end of the specimen. This opens the question of whether such behaviour can be caused by the initial presence of inhomogeneities or defects in that region of the specimen. It is known that defective regions can act as stress concentrators and thus could explain the unexpected behaviour of the material. For the biaxial mode 2, we observed a strain distribution that resembles the profile observed for uniaxial loading but with lower strain concentration at the boundaries of the specimen.

As described by Windle (1985 ▸), when tension is applied to PC its chains align preferentially towards the applied force. For this reason, we present the variation of the preferred orientation derived from peak C for PC as a function of scanning position to create a 2D mapping of the microstructural modification during the loading cycles. As shown in Fig. 5 ▸, the preferred orientation from peak C increases with increasing strain, and thus, the mapping shows a good one-to-one correlation of preferred alignment between the DIC data and the OI analysis. We also observed a strong correlation between strain and preferred orientation for peak B (and peak C), and to lesser extent for peak A and peak D (see Fig. S1 of supporting information). The orientation of the PC chains also showed an angular distribution towards the resulting load. Furthermore, during our experiments, we did not observe any systematic change in the SAXS signal that could indicate the formation of new structures or voids. The summarized results in Fig. 5 ▸ highlight the completeness of the data that can be acquired by the combination of full-field methods and SAXS/WAXS data.

**Figure 6:**
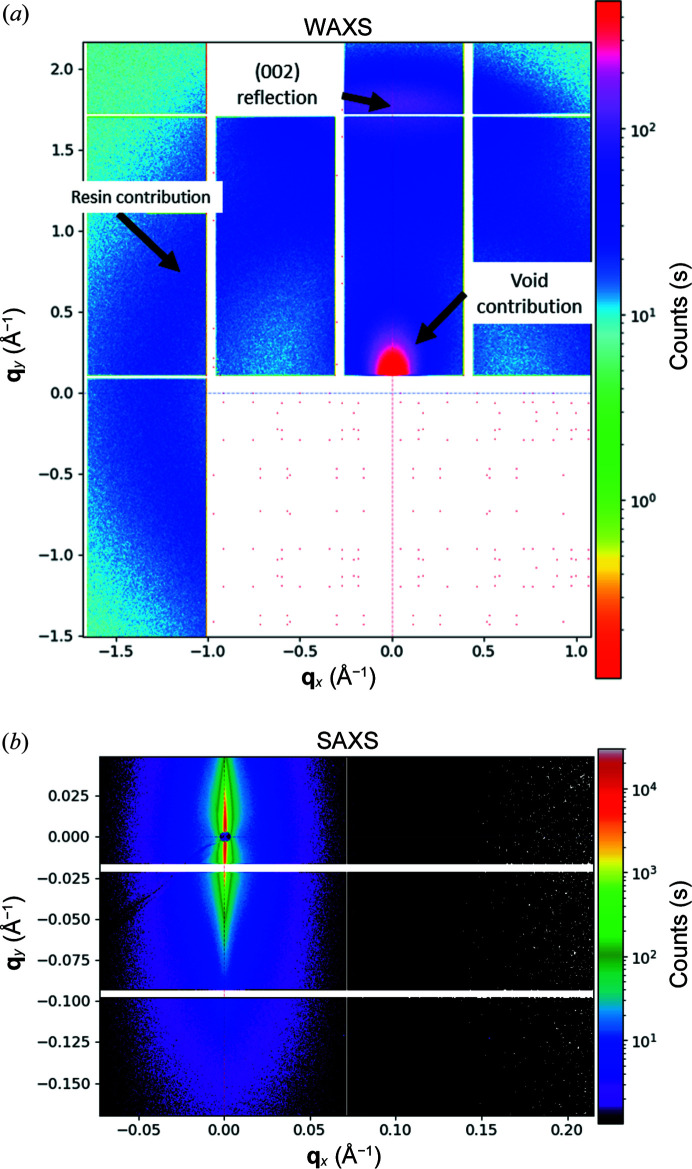
(*a*) 2D WAXS and SAXS scattering patterns of small CF bundles. The WAXS pattern is characterized by the diffraction peak from the crystallographic structure, accompanied by an isotropic ring due to the ep­oxy resin used as matrix, and the fan-shaped contribution from the presence of voids. (*b*) The features of the corresponding SAXS scattering pattern are mainly due to the presence of voids.

CFs are well known for their high tensile strength, high modulus and relatively low density (Frank *et al.*, 2012 ▸; Chung, 1994 ▸; Huang, 2009 ▸). CFs typically exhibit fibrillated structures, formed by small coherent domains or crystallites, amorphous regions, and a dispersion of defects or voids. The crystalline domains exhibit a regular hexagonal pattern that resembles that of 2D graphite, and thus, they are referred to as graphitic structures (Chung, 1994 ▸). The crystallographic microstructure is preferentially oriented towards the fibre axis, resulting in the intense signal observed in the vertical direction in Fig. 6 ▸(*a*). The characteristic information of the anisotropic 2D WAXS pattern was extracted along the equatorial direction with respect to the CF axis, along the meridional direction or parallel to the fibre axis, and in the azimuthal direction. Along the equatorial direction, the scattering curve was fitted with a pseudo-Voigt function. After considering the corresponding instrumental broadening, the apparent crystallite height or stacking of graphene planes was determined using the integral breadth of the (002) peak and the Scherrer equation. The preferred orientation of the graphene crystallites was estimated along the azimuthal direction at *q*_(002)_ considering a Gaussian function. The theoretical Young modulus was calculated using the relationship reported by Sauder & Lamon (2005 ▸) considering the microstructure parameters of CFs.

The irregular contour of the crystallites is believed to favour the creation of long and narrow voids between the carbon layers, resulting in a fan-shaped scattering pattern (Thünemann & Ruland, 2000 ▸ *a*, ▸ *b*). The fan-shaped pattern can be observed in the SAXS region [Fig. 6 ▸(*b*)]. Though the relation between the microstructure parameters and the Young modulus is well known, it is still under debate which parameters drive the strength properties, the development of fatigue and the failure process (Reynolds & Sharp, 1974 ▸; Sauder & Lamon, 2005 ▸).

The combination of SAXS/WAXS provides information on structural changes over a very broad range of length scales simultaneously, preventing variations of experimental conditions such as illumination area, collimation, beam energy or repeatability. For this reason, we carried out static loading on small CF bundles (tow size of 1k). The experimental conditions allowed us to map the changes in the orientation of the graphitic crystallites, as well as any variations in stacking or the development of stacking faults while providing information on the presence and evolution of voids.

For CFs, the relation between the crystallographic microstructure and their corresponding Young modulus is well known and arises from the elastic constants of the polycrystalline graphite and the preferred orientation to the fibre axis (Bennett *et al.*, 1983 ▸; Sauder & Lamon, 2005 ▸). Nevertheless, the relation between microstructure and tensile strength or specimen failure is still under discussion. When load is applied to a CF, its Young modulus also increases, a phenomenon known as elastic stiffening, as the crystallite domains rotate towards the fibre axis. The role of voids in the reorientation of the graphitic crystallites has been proposed as inhibition of crystalline mobility.

**Figure 7:**
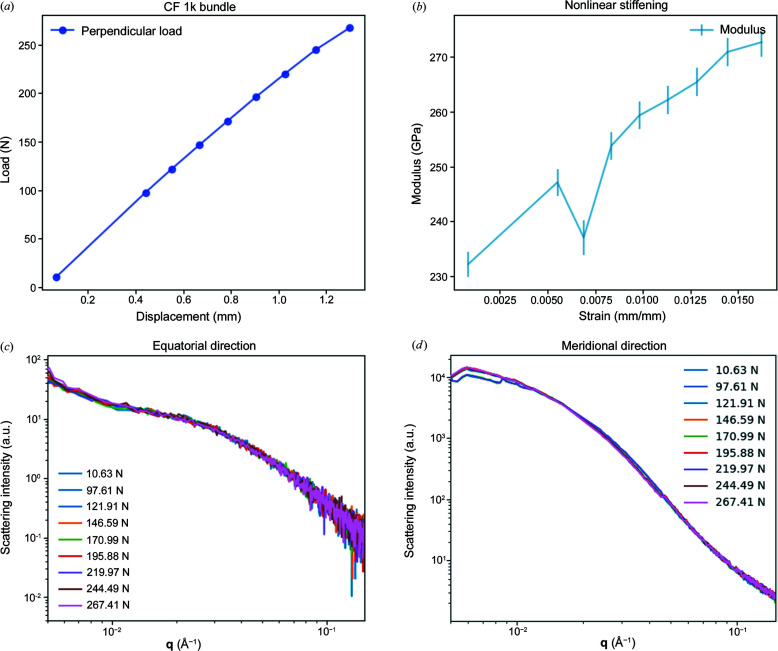
(*a*) Stress–strain curve for a small CF bundle (tow size of 0.4k) and (*b*) the corresponding modulus versus strain, considering a cross section of 0.8 mm^2^ and a bundle length of 80 mm. SAXS scattering intensity along the (*c*) equatorial and (*d*) meridional directions as a function of the scattering vector magnitude for each loading step.

Figs. 7 ▸(*a*) and 7 ▸(*b*) show the load versus displacement curve for the CF bundles and the determined modulus as a function of the engineering strain. The static loading was carried out in load control mode. We observe the increment of the Young modulus as the crystallites aligned further with increasing load. However, we did not observe any variation in the load versus displacement curve that indicated fatigue or failure. All bundles broke when approximately 300 N load was achieved at the grip area.

One of the proposed mechanisms behind the failure of CFs suggested that crystallite mobility can promote the combination of smaller crystallite regions into larger crystalline zones. In combination with improved alignment of the graphitic planes towards the fibre axis and strain, the conditions can promote planar mobility due to weak van der Waals interactions (Meek & Penumadu, 2021 ▸). However, we did not observe a significant increase in the crystallite dimensions using WAXS. Furthermore, it is expected that, as crystallite zones grow, defects such as voids appear too. Some authors have suggested that larger voids, while facilitating crystallite mobility, would also act as stress concentrators, leading to failure. From the SAXS measurements, we looked at the integrated scattering intensity of the void region. As a first estimation on this example, we did not observe a significant variation of the scattering intensity and/or the shape of the curve [see Figs. 7 ▸(*c*) and 7 ▸(*d*)]. As the scattering intensity is proportional to the square of the volume of the voids, if voids are created, we would observe a variation in the scattering intensity, as well as dependency with scattering angle. Typical void dimensions reported for PAN-based CFs range between 3 and 10 nm in length, and about 0.5 and 3 nm in width (Meek & Penumadu, 2021 ▸; Xu *et al.*, 2023 ▸). Nevertheless, future experiments involving cyclic loading of CF bundles with an experimental configuration that favours the detection of the scattering signal of features a few nanometres in size could potentially help us to determine whether the void dimensions are affected and recoverable when unloading.

## 4. Conclusions

We have presented a novel device for *in situ* biaxial deformation combined with small- and wide-angle X-ray scattering and DIC. The design allows a fast scan and high spatial resolution (beam size of 135 × 135 µm FWHM, horizontal × vertical, and translation resolution within 2 µm in the *X* and *Y* directions). Synchronization of the image acquisition with the detector triggering has been carried out for optimal correlation between the strain field and the corresponding scattering patterns, while the small beam size provides local information on the nano- and micro-structural rearrangement occurring.

The device allows us to explore new avenues of multi-axial deformation, which is of great importance to understand the mechanical behaviour of materials during more realistic loading conditions compared with pure uniaxial loading. The information gained from these multi-axial and multi-scale measurements is important for understanding material behaviour and a great foundation when developing physically based constitutive models.

## Supplementary Material

Supporting figures. DOI: 10.1107/S1600576723005034/uu5003sup1.pdf
