# Supplementary material for: In situ biaxial loading and multi-scale deformation measurements of nanostructured materials at the CoSAXS beamline at MAX IV Laboratory
Source: J Appl Crystallogr. 2023 Jun 30;56(Pt 4):967–75. doi: 10.1107/S1600576723005034 (PMC10405599; doi:10.1107/S1600576723005034)
Supplement: Supplementary file 1 [file j-56-00967-sup1.pdf]

JOURNAL OF
APPLIED
CRYSTALLOGRAPHY

Volume 56 (2023)

Supporting information for article:

In situ biaxial loading and multi-scale deformation measurements of nanostructured materials at the CoSAXS beamline at MAX IV Laboratory

Pablo Mota-Santiago, Jonas Engqvist, Stephen Hall, Roberto Appio, Maxime Maghe, Gautham Sathikumar, Matti Ristinmaa and Tomas S. Plivelic

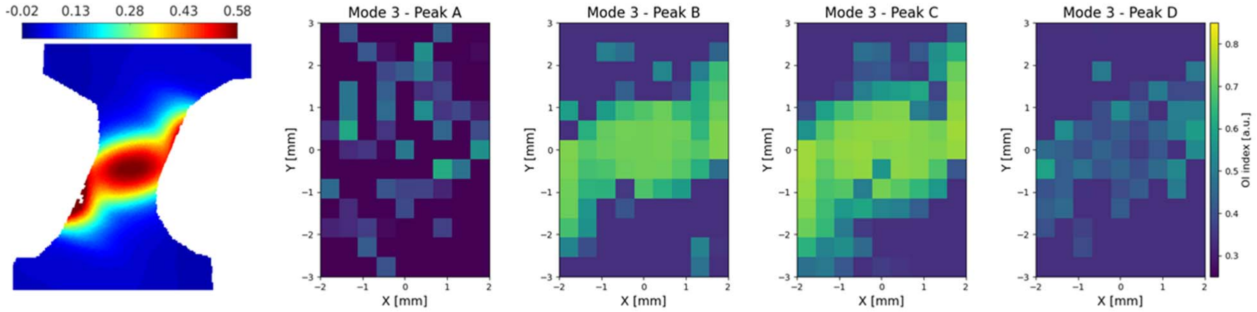

Figure S1 Full-field image of PC under deformation upon biaxial conditions (mode 3) and the corresponding spatial mapping of the OI index for from Peak A, B, C and D, respectively.
